# Effect of cryoprecipitate transfusion therapy in patients with postpartum hemorrhage: a retrospective cohort study

**DOI:** 10.1038/s41598-021-97954-5

**Published:** 2021-09-16

**Authors:** Ryo Kamidani, Takahito Miyake, Hideshi Okada, Genki Yoshimura, Keigo Kusuzawa, Tomotaka Miura, Ryuichi Shimaoka, Hideaki Oiwa, Fuminori Yamaji, Yosuke Mizuno, Ryu Yasuda, Yuichiro Kitagawa, Tetsuya Fukuta, Takuma Ishihara, Tomomi Shiga, Haruka Okamoto, Masahito Tachi, Masato Shiba, Norihide Kanda, Sho Nachi, Tomoaki Doi, Takahiro Yoshida, Shozo Yoshida, Kenichiro Morishige, Shinji Ogura

**Affiliations:** 1 Advanced Critical Care Center, Gifu University Hospital, 1-1 Yanagido, Gifu, 501-1194 Japan; 2 Department of Obstetrics and Gynecology, Gifu University Hospital, Gifu, Japan; 3 Innovative and Clinical Research Promotion Center, Gifu University Hospital, Gifu, Japan

**Keywords:** Medical research, Statistics

## Abstract

To evaluate the effect of cryoprecipitate (CRYO) transfusion in women referred for postpartum hemorrhage (PPH). This retrospective cohort study included patients with primary PPH referred to Gifu University Hospital between April 2013 and March 2020. We analyzed the effect of CRYO transfusion on fluid balance 24 h after the initial examination using a multivariable linear regression model adjusted for several confounding variables. To evaluate whether outcomes were modified by active bleeding, an interaction term of CRYO*active bleeding was incorporated into the multivariable model. We identified 157 women: 38 in the CRYO group (cases) and 119 in the control group. Fluid balance in the aforementioned period tended to decrease in the CRYO group compared with that in the control group (coefficient − 398.91; 95% CI − 1298.08 to + 500.26; p = 0.382). Active bleeding on contrast-enhanced computed tomography affected the relationship between CRYO transfusion and fluid balance (p = 0.016). Other outcomes, except for the overall transfusion requirement, were not significantly different; however, the interaction effect of active bleeding was significant (p = 0.016). CRYO transfusion may decrease the fluid balance in the first 24 h in PPH patients, especially in those without active bleeding.

## Introduction

Primary postpartum hemorrhage (PPH), especially critical obstetrical hemorrhage, involves life-threatening excessive bleeding during or after birth. Several studies have shown that fibrinogen levels drop early and significantly in PPH. PPH occurs in about 5% of pregnant women, and 80% of PPH cases are caused by atonic bleeding. Although PPH incidence has decreased due to improvements in perinatal and emergency medicine, it remains a major cause of maternal mortality and morbidity worldwide^1,2^. The goal of early blood transfusion therapy, used in combination with uterine artery embolization, bimanual compression of the uterus, and intrauterine tamponade, is hemostasis of minor vessels by increasing fibrinogen levels, which is a critical coagulation factor. Although early fibrinogen replacement therapy has gained support in recent years, current guidelines only recommend replacing fibrinogen when the plasma fibrinogen level drops to < 2 g/L during ongoing PPH or if the patient has received a massive blood transfusion^3,4^. The effects of fibrinogen replacement therapy on the total amount of blood transfusion or infusion fluids and mortality in the context of PPH remain unclear.

There are three dosage forms of fibrinogen: fresh frozen plasma (FFP), fibrinogen concentrate, and cryoprecipitate (CRYO). CRYO is the concentrated precipitate produced after thawing and centrifugation of FFP. It contains fibrinogen and other coagulation proteins such as factor VIII, factor XIII, von Willebrand factor, and fibronectin. CRYO has become an increasingly popular formulation in the treatment of PPH in recent years because it can increase fibrinogen levels without excessive fluid administration^5^. Compared with that for FFP, only about one-thirtieth the volume of CRYO is required to increase fibrinogen levels. CRYO transfusion is beneficial for hemostasis and is also advantageous over fibrinogen concentrates because it contains not only fibrinogen but also other coagulation factors. Its disadvantages are the same as those of FFP or fibrinogen concentrate, which include prolonged time to administration because of the need to thaw the product (CRYO is stored at temperatures <  − 25 °C), blood-related infections, transfusion-related lung injury, and allergic reactions^5,6^. However, CRYO melts and can be infused relatively quicker than FFP owing to the small total volume.

Matsunaga S et al. reported that administration of fibrinogen concentrates in massive obstetric hemorrhage cases increased the rate of fibrinogen supplementation fivefold and reduced the FFP dosage, the FFP/red cell concentrate (RCC) ratio, and the incidence of pulmonary edema^7^. To the best of our knowledge, it is unclear whether CRYO administration as fibrinogen replacement therapy improves mortality or inhibits fluid overload. To test the hypothesis that CRYO use decreases the transfusion or infusion requirement and improves mortality when applied as fibrinogen replacement therapy, we performed this study to compare outcomes among patients who underwent early fibrinogen replacement therapy with and without CRYO.

## Results

### Baseline characteristics

**Figure 1:**
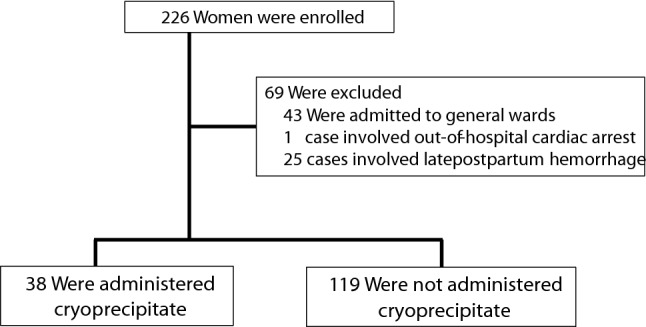
Flowchart of participant enrolment in the study.

**Figure 2:**
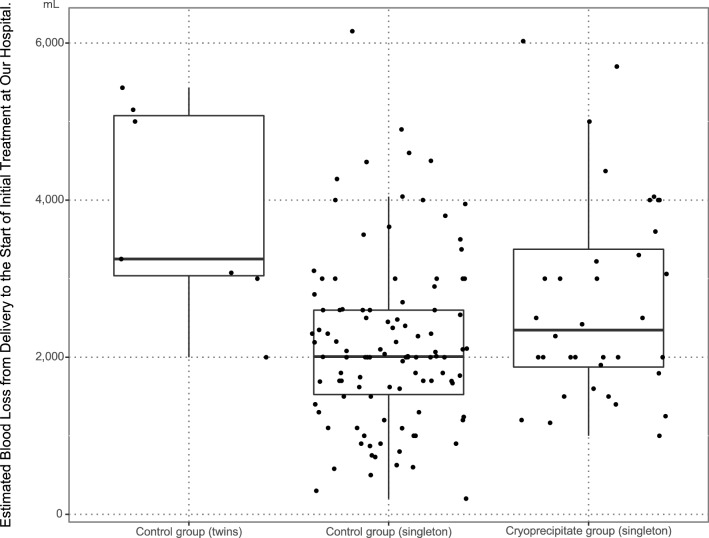
Estimated blood loss from delivery to the start of initial treatment at our hospital.

Between April 2013 and March 2020, a total of 226 women with PPH were enrolled (Fig. 1). The analysis included 157 eligible participants who met the criteria of this study: 38 in the CRYO group (cases) and 119 in the control group (controls). Table 1 summarizes the patients’ clinical characteristics. Age, gestational age, parity, mode of delivery, transfer time, transfusion before arrival, body temperature, and use of assisted reproductive technology (ART) did not differ significantly between the two groups. The shock index, prothrombin time-international normalized ratio (PT-INR), Acute Physiology and Chronic Health Evaluation II (APACHE II) score, Clark’s criteria score, obstetric disseminated intravascular coagulation (DIC) score, incidence of transfusion before arrival, major pregnancy complications, and lactate values were higher in the CRYO group than in the control group. The values of other blood parameters (hemoglobin, fibrinogen, and platelet count) were lower in the CRYO group than in the control group. Figure 2 shows the estimated blood loss from delivery to the start of initial treatment at our hospital; only three groups are shown because there were no twin pregnancies in the CRYO group. The number of patients who met each diagnostic criterion and were diagnosed with obstetric DIC was 19 (50.0%) in the CRYO group and 10 (8.4%) in the control group based on Clark’s criteria, and nine (23.7%) in the CRYO group and five (4.2%) in the control group for the obstetric DIC score. Of the patients diagnosed with obstetric DIC with a Clark’s criteria score of 3 or more, eight (88.9%) in the CRYO group and five (100.0%) in the control group were weaned from obstetric DIC after 24 h.

**Table 1:** Clinical characteristics of the participants.

Variable	N	Overall, N = 157^1^	Control group, N = 119^1^	CRYO group, N = 38^1^	p-value^2^
Age, yrs	157	34 (30, 37)	34 (30, 37)	34 (30, 38)	0.632
Gestational age, wk	155	39.14 (37.71, 40.29)	39.29 (37.86, 40.29)	38.57 (37.43, 39.86)	0.276
Parity	153				0.940
1		93 (60.8%)	72 (61.5%)	21 (58.3%)	
2		42 (27.5%)	31 (26.5%)	11 (30.6%)	
3		17 (11.1%)	13 (11.1%)	4 (11.1%)	
> 4		1 (0.7%)	1 (0.9%)	0 (0%)	
Singleton/multiple birth	157				0.200
Singleton		149 (94.9%)	38 (100.0%)	111 (93.3%)	
Twin		8 (5.1%)	0 (0.0%)	8 (6.7%)	
Mode of delivery	157				0.127
Vaginal delivery		96 (61.1%)	77 (64.7%)	19 (50.0%)	
Caesarean section		61 (38.9%)	42 (35.3%)	19 (50.0%)	
Transfer time, min	127	208.0 (115.0, 333.5)	222.0 (117.0, 335.0)	197.5 (95.8, 316.8)	0.489
Transfusion before arrival	157	31 (19.7%)	17 (14.3%)	14 (36.8%)	0.004
Using of ART	139	67 (48.2%)	54 (51.4%)	13 (38.2%)	0.236
Major pregnancy complications	156	12 (7.7%)	6 (5.1%)	6 (15.8%)	0.072
Lactate, mg/dL	153	25.0 (17.0, 34.0)	23.0 (15.0, 31.2)	30.0 (21.0, 40.0)	0.009
Hemoglobin, g/dL	157	7.4 (6.0, 9.3)	7.6 (6.3, 9.4)	6.6 (5.2, 8.0)	0.026
Fibrinogen, mg/dL	155	183.0 (136.5, 265.5)	214.0 (150.0, 285.0)	140.0 (53.8, 178.8)	< 0.001
PT-INR	153	1.1 (1.0, 1.2)	1.1 (1.0, 1.2)	1.2 (1.1, 1.4)	< 0.001
Platelet count, × 10^3^/μL	157	134.0 (104.0, 182.0)	140.0 (115.0, 193.5)	95.0 (49.0, 148.2)	< 0.001
Body temperature, °C	157	36.7 (36.3, 37.1)	36.6 (36.3, 37.0)	36.8 (36.0, 37.2)	0.805
Shock Index	157	0.9 (0.7, 1.2)	0.9 (0.7, 1.2)	1.1 (0.8, 1.4)	0.038
APACHE II score, pts	156	11.0 (9.0, 14.0)	11.0 (9.0, 13.0)	13.0 (10.0, 18.0)	0.001
Clark’s criteria, pts	157	1.0 (0.0, 2.0)	0.0 (0.0, 1.0)	2.5 (1.0, 3.0)	< 0.001
Number of patients who met Clark’s DIC criteria (≥ 3 scores)	157	29 (18.5%)	10 (8.4%)	19 (50.0%)	< 0.001
Obstetrics DIC score, pts	156	6.0 (4.0, 9.0)	5.0 (3.0, 8.0)	9.0 (7.0, 12.0)	< 0.001
Number of patients who met obstetrics DIC score (≥ 13 scores)	156	14 (9.0%)	5 (4.2%)	9 (23.7%)	0.001

Major pregnancy complications include pregnancy hypertension syndrome, gestational diabetes, and abruptio placentae. Body temperature was recorded as the lowest body temperature during blood transfusion. Shock index was calculated as heart rate divided by systolic blood pressure.

CRYO, cryoprecipitate; BMI, body mass index; ART, assisted reproductive technology; APACHE II score, Acute Physiology and Chronic Health Evaluation II score; DIC, disseminated intravascular coagulation.

^1^Statistical data are presented as the median (interquartile range) or n (%).

^2^Statistical tests performed: Wilcoxon rank-sum test; Fisher's exact test.

### Outcomes

**Figure 3:**
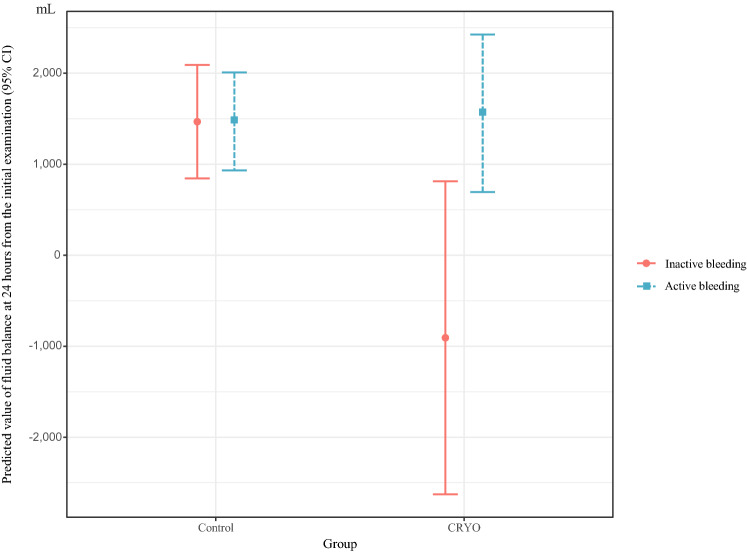
Association between use of CRYO and fluid balance at 24 h from the initial examination by presence of active bleeding. CI, confidence interval. CRYO, cryoprecipitate.

Tables 2 and 3 present the outcome variables of our study. Although the fluid balance at 24 h from the initial examination was not significantly different between the two groups, it tended to be lower in the CRYO group than in the control group as follows: mean: + 1544 mL (range, − 4036 to + 7307 mL) vs. + 1225 mL (range, − 1958 to + 7348 mL); coefficient: − 398.91; 95% CI: − 1298.08 to + 500.26; p = 0.382). However, fluid balance at 24 h from the initial examination differed significantly in the presence of active bleeding (p = 0.016). The adjusted mean fluid balance was + 1467.74 mL (95% CI 844.25 to 2091.24) in the no active bleeding/control group, − 907.26 mL (95% CI − 2627.18 to 812. 67) in the no active bleeding/CRYO group, + 1470.1 mL (95% CI 931.76 to 2008.44) in the active bleeding/control group, and + 1560.91 mL (95% CI 694.89 to 2426.94) in the active bleeding/CRYO group (Fig. 3).

**Table 2:** Multivariable linear regression analysis.

Outcome	N	Control group, N = 119^1^	CRYO group, N = 38^1^	Coefficient	95%LCI	95%UCI	p-value^2^
Overall transfusion requirement, units	157			701.64	158.58	1244.7	0.012
RCC		4 (1, 6)	8 (6, 14)				
FFP		2 (0, 6)	8 (4, 17)				
PC		0 (0, 0)	0 (0, 20)				
Total infusion volume within 24 h, mL	157	3904 (3067, 5043)	5033 (4413, 6793)	1.16*	0.99	1.37	0.074
IN–OUT balance within 24 h, mL	157	1225 (232.5, 2384)	1550 (256, 2950)	− 398.91	− 1298.08	500.26	0.382
Obstetrics DIC score variation, pts	157	− 2 (− 1, − 5)	− 7 (− 3, − 10.5)	− 0.2	− 0.89	0.49	0.568

CRYO, cryoprecipitate; RCC, red cell concentrate; FFP, fresh frozen plasma; PC, platelet concentrate; ICU, intensive care unit; DIC, disseminated intravascular coagulation.

^1^Statistical data are presented as the median (interquartile range).

^2^Statistical tests performed: multivariable linear regression.

*Coefficients obtained from multivariable linear regression analysis for log-transformed outcome values are back-transformed to the original scale. This coefficient indicates the fold-increase of the outcome in the Cryoprecipitate group relative to the Control group.

**Table 3:** Multivariable binary/proportional odds logistic analysis.

Outcome	N	Control group, N = 119^1^	CRYO group, N = 38^1^	Odds ratio	95%LCI	95%UCI	p-value^2^
Transcatheter arterial embolization	157	71 (59.7%)	31 (81.6%)	1.01	0.08	12.53	0.993
Hospital stay, days	157	4 (3, 6)	5 (4, 9.5)	1.46	0.69	3.11	0.323
Ventilator stay, days	157	0 (0, 0)	0 (0, 0)	8.32	0.96	71.79	0.054
ICU stay, days	157	2 (2, 3)	3 (2, 3)	1.32	0.54	3.2	0.544

CRYO, cryoprecipitate; ICU, intensive care unit.

^1^Statistical data are presented as the median (interquartile range) or n (%).

^2^Statistical tests performed: multivariable logistic regression; multivariable proportional odds logistic regression.

The overall transfusion requirement was significantly higher in the CRYO group than in the control group: mean: 2390 mL (range, 340 to 13,660 mL) vs. 840 mL (range, 0 to 4640 mL); coefficient: + 701.64 mL; 95% CI: + 158.58 to + 1244.7 mL; p = 0.012). The other outcomes were not significantly different between the two groups. In the CRYO and control groups, respectively, other outcomes included hysterectomy in four (10.5%) and zero (0%) patients, neurological complications in one (2.6%) and zero (0%), and 28 – day mortality in zero (0%) and zero (0%) patients.

## Discussion

This study highlights the use of CRYO for PPH. CRYO tended to reduce the fluid balance 24 h from the initial examination, although the difference was not statistically significant. In a sub-analysis, the use of CRYO significantly reduced the fluid balance at 24 h from the initial examination in those without active bleeding.

An ability to decrease the fluid balance has clinical value. This decrease reduces the risk of congestive heart failure, acute kidney injury, prolonged ventilator use, and mortality^8–10^. In recent years, several studies have shown that fluid overload causes endothelial glycocalyx (eGCX) injury and results in the extravasation of fluid^11,12^. The sugar–protein glycocalyx coats the entirety of healthy vascular endothelial cells and plays a key role in microvascular and endothelial physiology through its influence on the regulation of microvascular tone and endothelial permeability and by inhibiting intravascular thrombosis^13^. The eGCX is reportedly damaged under stressful conditions such as surgical invasion, systemic inflammation including sepsis, and severe diseases such as acute kidney injury, chronic kidney disease, and cardiovascular disease^14–18^. In the recently proposed revised Starling Principle, microvascular fluid exchange flow is defined by the collagen osmotic pressure in the intercellular space below the eGCX and not by intercellular substances^19^. According to this principle, a rapid increase in the hydrostatic pressure of the capillary cavity due to fluid overload increases the fluid exchange flow and causes eGCX injury, leading to further extravascular leakage.

Resuscitation with FFP transfusion in the context of hemorrhagic shock preserves the glycocalyx thickness in comparison with resuscitation with normal saline, lactated Ringer’s solution, or 5% albumin in rats^20^. CRYO administration contributes to the maintenance of eGCX thickness. Reducing the fluid balance in the hyperacute phase of PPH can reduce eGCX injury caused by fluid overload; this can consequently reduce systemic edema and the exacerbation of a hemodynamic state. We believe that CRYO is especially beneficial for patients without active bleeding. In this study, patients without active bleeding had a significantly negative fluid balance in the hyperacute phase, and transfusion therapy could be a major treatment strategy for hemodynamic stabilization. Zaidi et al. published a systematic review of the early use of fibrinogen replacement therapy in PPH patients, which included two completed randomized control studies (RCT) and no non-randomized studies^5^. Both RCTs were underpowered to detect differences in any of the outcomes, including transfusion requirements. The effect of CRYO transfusion as the replacement method has not been studied in a completed RCT. Green et al. reported a protocol for a study investigating the effect of early CRYO transfusion versus standard care in women who developed severe postpartum hemorrhage (ACROBAT). This is the only registered and ongoing RCT on the use of CRYO for PPH^21^.

This study has some limitations. First, our observations were limited to a relatively small population, and a larger data set should be studied in the future. Therefore, we could not adjust for biases associated with disease severity. Second, the groups were not randomized, and CRYO use was a part of some transfusion protocols. We found that the overall transfusion requirement in the CRYO group was significantly increased in this study. This might have been influenced by the use of CRYO in patients needing larger transfusion volumes. Additionally, there may have been biases related to the management protocols used before referral in primary maternity hospitals and after arrival in our hospital. Transfusion strategies are physician-led, and related adjustment was beyond the scope of this study. Basically, the criteria for administering CRYO are at the discretion of each physician, and it is acceptable to start administering CRYO before confirming the blood test results, depending on a patient’s hemodynamics and shock index. Therefore, we have not been able to examine the classification of groups according to coagulation factors.

In conclusion, to our knowledge, no study has reported that CRYO use significantly decreases the transfusion or infusion requirement in PPH patients. Our study suggests that CRYO use could help reduce transfusion or infusion volume requirements. This may improve clinical outcomes such as reducing the need a ventilator or the duration of an intensive care unit (ICU) stay. We propose that an active transfusion therapy including CRYO is more effective in PPH patients without active bleeding. Future studies on PPH should formulate a transfusion strategy involving CRYO for patients who benefit from this approach and establish other multi-disciplinary strategies, including interventional angiography.

## Materials and methods

### Study oversight and design

We carried out a retrospective, single-center cohort study involving women with PPH who were referred to the Advanced Critical Care Center, Gifu University Hospital, between April 2013 and March 2020. This study was approved by the institutional ethics committees of Gifu University (approval #2020–170, approved on November 4, 2020). The institutional ethics committees of Gifu University Graduate School of Medicine approved the substitution of an opt-out notice for informed consent from patients because of the retrospective nature of the study, the design of which was based on computerized data with anonymous selection.

### Study patients

We included women aged 18 years or older with PPH who were referred to our tertiary care center. The criteria for referral were dependent on each previous physician, in addition to continuous hemorrhage with abnormal vital signs, a high shock index (≥ 1.5), or DIC. The shock index was calculated as the heart rate divided by systolic blood pressure. We excluded cases of cardiopulmonary arrest on arrival, general ward hospitalization, and late PPH (defined as PPH occurring more than 24 h after delivery) from our analysis.

### Definitions

The clinical diagnostic definition of PPH varies from country to county. It has been reported that the 90th percentile values for volumes of blood loss experienced as a result of hemorrhage during delivery in Japan are 800 mL in singleton vaginal delivery and 1600 mL in twin vaginal delivery. The reported volume, including amniotic fluid, is 1500 mL for singleton and 2300 mL for twins in the cesarean section^22^. In this study, the inclusion criteria were cases that were diagnosed as PPH by the previous obstetrician and gynecologist prior to transfer to our hospital, regardless of whether they were singleton or twin births. We defined DIC in obstetrics as a DIC Diagnostic Criteria in Obstetrics score of 13 or higher and Clark’s criteria score of 3 or higher. The DIC Diagnostic Criteria in Obstetrics refer to a scoring system that does not place a great deal of weight on the blood test results for starting early treatment. It includes underlying prenatal problems, clinical symptoms, and blood test results (Table 4)^23^. Clark et al. also reported a diagnostic criterion for obstetric DIC using clinical data of PT, platelet counts, and fibrinogen levels^24^. We defined major pregnancy complications as pregnancy hypertension syndrome, gestational diabetes, and abruptio placentae. Active bleeding was defined as existing extravasation on contrast-enhanced computed tomography. In our hospital, the goal of transfusion therapy for PPH is to maintain a hemoglobin level > 8.0 g/dL and a fibrinogen level > 200 mg/dL. RCC and FFP are administered as the initial transfusion product at a ratio of 1:1 or higher; additionally, 15 units of CRYO are administered per transfusion. The decision to administer CRYO in addition to FFP is left to the discretion of the physician. If the patient exhibits hemodynamic instability, CRYO can be administered without waiting for blood test results based on the amount of estimated blood loss and the shock index. In case of emergency transfusions, 10 units of RCC, 10 units of FFP, and 15 units of CRYO are ordered for immediate administration.

**Table 4:** DIC diagnostic criteria in obstetrics.

	Score
1. Underlying diseases	
a. Placental abruption	
Stiffening of the uterus, death of the fetus	5
Stiffening of the uterus, survival of the fetus	4
Confirmatory diagnosis of placental abruption by ultrasonic tomographic findings and CTG findings	4
b. Amniotic fluid embolism	
Acute cor pulmonale	4
Artificial ventilation	3
Assisted respiration	2
Oxygen flux alone	1
c. DIC-type postpartum hemorrhage	
In case the blood from the uterus has low coagulability	4
Hemorrhage of 2000 mL ≦ (within 24 h after the start of hemorrhage)	3
Hemorrhage of 1000 mL ≦, but not exceeding 2000 mL (within 24 h after the start of hemorrhage)	1
d. Eclamptic attack	4
e. Severe infection	
Those with fever accompanied by shock, bacteremia, and endotoxemia	4
Continued fever or remittent fever	1
f. Other underlying diseases	1
2. Clinical symptoms	
a. Acute renal failure	
Anuria (≦5 mL/h)	4
Oliguria (5–20 mL/h)	3
b. Acute respiratory failure (amniotic fluid embolism excluded)	
Artificial ventilation or occasional assisted respiration	4
Oxygen flux alone	1
c. Organ failure	
Heart (rales or foamy sputum, etc.)	4
Liver (visible jaundice, etc.)	4
Brain (clouding of consciousness, convulsion, etc.)	4
Digestive tract (necrotic enteritis, etc.)	4
Other severe organ failure	4
d. Hemorrhage diathesis (Macroscopic hematuria and melena, purpura, hemorrhage from the mucous membrane, gingival bleeding, bleeding at the site of injection, etc.)	4
e. Shock symptoms	
Pulse rate ≧ 100/min	1
Blood pressure ≦ 90 mmHg(systolic) or blood pressure reduction of ≧ 40%	1
Cold sweat	1
Pallor	1
3. Laboratory findings	
Serum FDP ≧ 10 μg/mL	1
Platelet counts ≦ 10 × 10^4^ /μL	1
Fibrinogen ≦ 150 mg/dL	1
PT ≧ 15 (s) (≦50%) or hepaplastin test ≦50%	1
Erythrocyte sedimentation rate ≦4 mm/15 min or ≦15 mm/h	1
Bleeding time ≧ 5 min	1
Other coagulation and fibrinolysis factors; AT ≦18 mg/dL or ≦60%, prekallikrein, α2-PI, plasminogen, other coagulation factors ≦50%	1

CTG, cardiotocogram; DIC, disseminated intravascular coagulation; FDP, fibrinogen and fibrin degradation; PT, prothrombin time; AT, antithrombin.

### Data collection

We collected data from the electronic medical records on age, gestational age at delivery, parity, mode of delivery, transfer time, transfusion before arrival, use of ART, major pregnancy complications, blood test results, shock index, APACHE II score, Clark’s criteria score, and DIC Diagnostic Criteria in Obstetrics score. Parity includes the current delivery, and the first birth was calculated as parity “1.” The APACHE II system is a severity-of-disease classification system, one of several ICU scoring systems^25^. It is calculated within 24 h of admission to an ICU.

### Primary and secondary outcomes

The primary outcome of interest in this study was the fluid balance at 24 h from the initial examination, defined as the total out-volume (urine, excretion, and hemorrhage volume) subtracted from the in-volume (infusion and transfusion volume). We calculated this value from the medical chart and surgical records as the 24 h following the initial examination.

The secondary outcomes were the overall transfusion requirement, total infusion volume within 24 h, transcatheter arterial embolization, hysterectomy, durations of ICU and hospital stays and ventilator use, and the Efficacy Evaluation Criteria for DIC in Obstetrics score. The Efficacy Evaluation Criteria for DIC in Obstetrics scores were based on combined improvements in clinical symptoms and blood test results. This system classifies patients as excellent, good, fair, poor or aggravated based on pre- and post-treatment scores (Table 5). We compared the Efficacy Evaluation Criteria for DIC in Obstetrics scores at arrival and after 24 h. We used the most recent results when blood test results were not available after 24 hours^26^.

**Table 5:** Efficacy evaluation criteria for DIC in obstetrics.

1. Clinical symptoms					
It is based on “2. Clinical symptoms” of DIC Diagnostic Criteria in Obstetrics (Table 1)					
2. Coagulation Tests					
Score	0	1	2	3	4
Serum FDP μg/mL	< 10	10 ≦ < 20	20 ≦ < 40	40 ≦ < 80	80≦
Platelet count × 10^4^/μL	20 <	16 < ≦ 20	12 < ≦ 16	8 < ≦ 12	≦8
Fibrinogen mg/dL	200 <	150 < ≦ 200	100 < ≦ 150	50 < ≦ 100	≦50
PT sec	12 <	12 ≦ < 15	15 ≦ < 20	20 ≦ < 25	25≦
3. Evacuation of efficacy (post–pre)					
	Excellent	Good	Fair	Poor	Aggravated
Difference	≦ − 9	− 9 < ~ ≦ − 5	− 5 < ~ ≦ − 1	± 0	+ 1≦

DIC, disseminated intravascular coagulation; FDP, fibrinogen and fibrin degradation; PT, prothrombin time.

### Sample size

The sample size in this study was determined based on the number of covariates included in the statistical model for the primary analysis and overfitting^27^ and based on data availability.

### Statistical analysis

Continuous data were described using the median and interquartile range, and categorical data were described using frequencies with proportions. To evaluate the effect of the use of CRYO on fluid balance at 24 h from the initial examination, we conducted a multivariate linear regression analysis adjusted for covariates, including age, hypertensive syndrome in pregnancy, fibrinogen levels, hemoglobin levels, shock index, lactic acid levels, active bleeding, APACHE II scores, and DIC Diagnostic Criteria in Obstetrics scores. The covariates were selected as potential confounders a priori based on previous studies^8,27–29^ and expert opinions from a physician in the field. Moreover, we incorporated a two-way interaction term (use of CRYO*active bleeding) to assess the effect of modification of active bleeding on the relationship between the use of CRYO and fluid balance at 24 h from the initial examination. We considered a modifying effect of active bleeding if the p-value of the global test for the interaction term was < 0.05.

We also evaluated the relationship between the use of CRYO and the secondary outcomes. The multivariable linear regression model was used for assessing the effect of CRYO use on the overall transfusion requirement, total infusion volume within 24 h, or the DIC score. We applied log transformation to the total infusion volume within 24 h and used that as the dependent variable in the regression model because the distribution was skewed. Multivariate logistic regression was performed to assess the relationship between CRYO use and transcatheter arterial embolization. We also employed multivariate proportional odds logistic regression to assess the relationship between CRYO use and the duration of hospital or ICU stays or ventilator use. All multivariate models used in the secondary analyses were adjusted for the same covariates as those in the primary analysis. A two-sided p-value < 0.05 was considered statistically significant^30^. All analyses were performed using R software (version 4.0.3; available at http://www.r-project.org)^30^.

### Ethics approval and consent to participate

The study conformed with the principles outlined in the Declaration of Helsinki^31^. Ethics approval was obtained from the institutional ethics committees of Gifu University Graduate School of Medicine (Approval #2020-170, approved on November 4, 2020). The institutional ethics committees of Gifu University Graduate School of Medicine approved the substitution of an opt-out notice for informed consent from patients because of the retrospective nature of the study, whose design was based on computerized data with anonymous selection.

## Consent for publication

Written informed consent was obtained from the patient for publication of this retrospective cohort study and any accompanying images..

## Data Availability

The data that support the findings of this study are available from the corresponding author, [TM], upon reasonable request.
